# Mesoporous Thin Film Architectures: Addressing Material Demands through Molecular Self-Assembly

**DOI:** 10.1021/accountsmr.5c00262

**Published:** 2025-12-02

**Authors:** Alberto Alvarez-Fernandez, Jon Maiz, Marco Faustini, Morgan Stefik, Galo J. A. A. Soler-Illia, Stefan Guldin

**Affiliations:** † Centro de Fisica de Materiales (CFM-MPC), CSIC-EHU, 20018 Donostia-San Sebastian, Spain; ‡ IKERBASQUE-Basque Foundation for Science, Plaza Euskadi 5, 48009 Bilbao, Spain; § 27063 Laboratoire Chimie de la Matière Condensée de Paris LCMCP, CNRS, Sorbonne Universite, 75005 Paris, France; ∥ Institut Universitaire de France (IUF), 75231 Paris, France; ⊥ Department of Chemistry and Biochemistry, 2629 University of South Carolina, Columbia, South Carolina 29208, United States; # Instituto de Nanosistemas, Escuela de Bio y Nanotecnología, UNSAM, CONICET, Avenida 25 de Mayo 1169, San Martín, Buenos Aires 1650, Argentina; 7 Department of Life Science Engineering, Technical University of Munich, Gregor-Mendel-Straße 4, 85354 Freising, Germany; 8 TUMCREATE, 1 CREATE Way, #10-02 CREATE Tower, 138602, Singapore; 9 Department of Chemical Engineering, 4919 University College London, Torrington Place, London WC1E 7JE, United Kingdom

## Abstract

Mesoporous thin films spark interest across a wide range of disciplines due to their tunable nanostructures, large internal surface areas, and strong compatibility with planar optical, electronic, and microfluidic devices. While attention in the porous materials community has shifted toward macroporous or disordered nanoporous systems, a resurgence in mesoporous thin film research is underway, driven by new molecular self-assembly methods, advanced materials chemistry, and improved characterization techniques. The integration of high-χN block copolymer design, kinetically persistent micelle templating, and postdeposition processing protocols now allows control over structural parameters such as pore size, wall thickness, porosity, and connectivity.

These advances have overcome many of the thermodynamic and processing constraints that previously limited widespread adoption. Rather than serving only as high-surface-area supports, mesoporous thin films are engineered as active interfaces where responsive chemistries and nanoscale confinement act in tandem. Embedding switchable ligands, thermoresponsive polymers, redox mediators, or ion-selective groups directly within the pore walls enables real-time control over transport, optical, and electrochemical properties. These capabilities open up new directions in adaptive coatings, gated membranes, and fast-response biosensors. To further expand their functional scope, mesoporous films are integrated into hierarchical and multicomponent architectures. Techniques such as triblock terpolymer templating, crack-directed assembly, and nanoimprint lithography allow for control over spatial organization on the micron and submicron scale and pore system orientation. This enables programmable anisotropy, enhanced molecular diffusion, and wavelength-selective photonic behavior, essential for next-generation sensing, catalysis, and energy applications.

Such structural and functional complexity requires equally sophisticated characterization. Multimodal and in situ techniques can track material dynamics under operational conditions. Recent progress includes extended-range ellipsometric porosimetry (EP) for hierarchical architectures, vacuum EP for interface energetics, time-resolved EP for diffusion kinetics, and correlative AFM-SAXS mapping. The introduction of advanced neutron-based spectroscopies, particularly quasielastic neutron scattering (QENS), promises to provide real-time access to ion transport dynamics and segmental motion under nanoscale confinement, offering a path toward deeper mechanistic understanding of structure-performance correlations in mesoporous systems.

This Account reflects the technical advances made and the interdisciplinary collaborations that have shaped our collective vision. The particular dimensions of mesopores enable us to subtly tune interactions at the molecular, interfacial, and mesoscopic levels that permit us to harness nanoconfinement. What emerges is a versatile, modular platform capable of chemical gating, energy transduction, and sensing with a level of tunability unmatched by other porous materials. We highlight critical challenges including the need for more robust large-area processing, a deeper understanding of dynamic behavior under cycling, and better integration with device-level architectures. Our strategies support the transition of mesoporous thin films into active high-performance components in next-generation energy, environmental, and biomedical systems.

## Graphical abstract

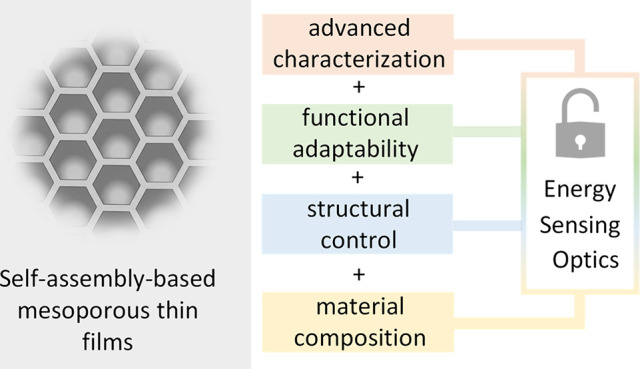

## 1 Introduction

Mesoporous thin films offer a uniquely versatile platform for integrating high internal surface areas, nanoscale confinement, and tunable surface chemistry into planar, device-compatible formats.^1^ Their potential for selective transport, chemical functionalization, and confined reactivity has long made them attractive candidates for applications in sensing,^2,3^ energy conversion and storage,^4^ optical coatings,^5^ and separation membranes.^6,7^ Their appeal lies not only in structural characteristics, but in the ease and reproducibility with which they can be modified to respond to chemical or physical stimuli.^8^

Yet translating this promise into thin-film formats introduced a distinct set of challenges. Early breakthroughs in bulk mesoporous materials, such as surfactant-templated MCM-41 ^9^ and the use of amphiphilic block copolymers for structural control,^10–12^ provided a strong foundation. However, adapting these strategies to thin films exposed complex interfacial effects, solvent evaporation dynamics, and competing kinetic and thermodynamic pathways that often compromised reproducibility and structural order.^14–16^ In parallel, application requirements evolved: fields like catalysis increasingly demanded microporous architectures with high site densities to enhance small-molecule reactivity, while emerging needs in energy and sensing required films with distinct electronic properties, larger, more connected pores, and orthogonal surface chemistry. These limitations narrowed the scope of mesoporous thin film applications, confining them to niche roles such as tunable refractive index coatings.

Over the past decade, a combination of synthetic and analytical breakthroughs has begun to change this picture. Advances in supramolecular design, selective reactivity, coassembly, and postprocessing techniques have unlocked new levels of control over pore size, wall thickness, and hierarchical structure.^13–16^ At the same time, advanced multimodal characterization, from ellipsometric porosimetry (EP) to atomic force microscopy (AFM) and small-angle X-ray scattering (SAXS) mapping, has enabled an enhanced understanding of structure, function, and performance.^17^ EP, in particular, has evolved from a pore-size profiling method into a more versatile and quantitative tool. Recent developments, including the use of high-molar-mass probe vapors and vacuum-based analysis, now enable nondestructive measurements of pores up to ∼ 80 nm, as well as the extraction of previously inaccessible parameters such as internal contact angles and surface energies.^18–20^

In this Account, we reflect on how recent advances, many emerging from our own laboratories, have helped transform mesoporous thin films from scientific curiosities into key enablers of device functionality. Rather than offering a comprehensive review, we highlight selected developments in materials design, synthesis, and device integration where our groups have played an active role in advancing the field. Figure 1 summarizes the four interconnected pillars that have driven the recent evolution of mesoporous thin films: (i) structural control enabled by soft-templating and supramolecular engineering; (ii) compositional flexibility across oxides, metals, and hybrids; (iii) functional adaptability through selective surface modification; and (iv) advanced characterization tools capable of probing both static structure and dynamic response. Together, these elements define a modular platform in which structure, chemistry, and function can be decoupled and reassembled, a key step toward programmable materials with application-specific performance. This Account aims to offer a coherent perspective on the evolving role of mesoporous thin films and what open challenges remain in translating them into fully functional, integrated systems.

**Figure 1:**
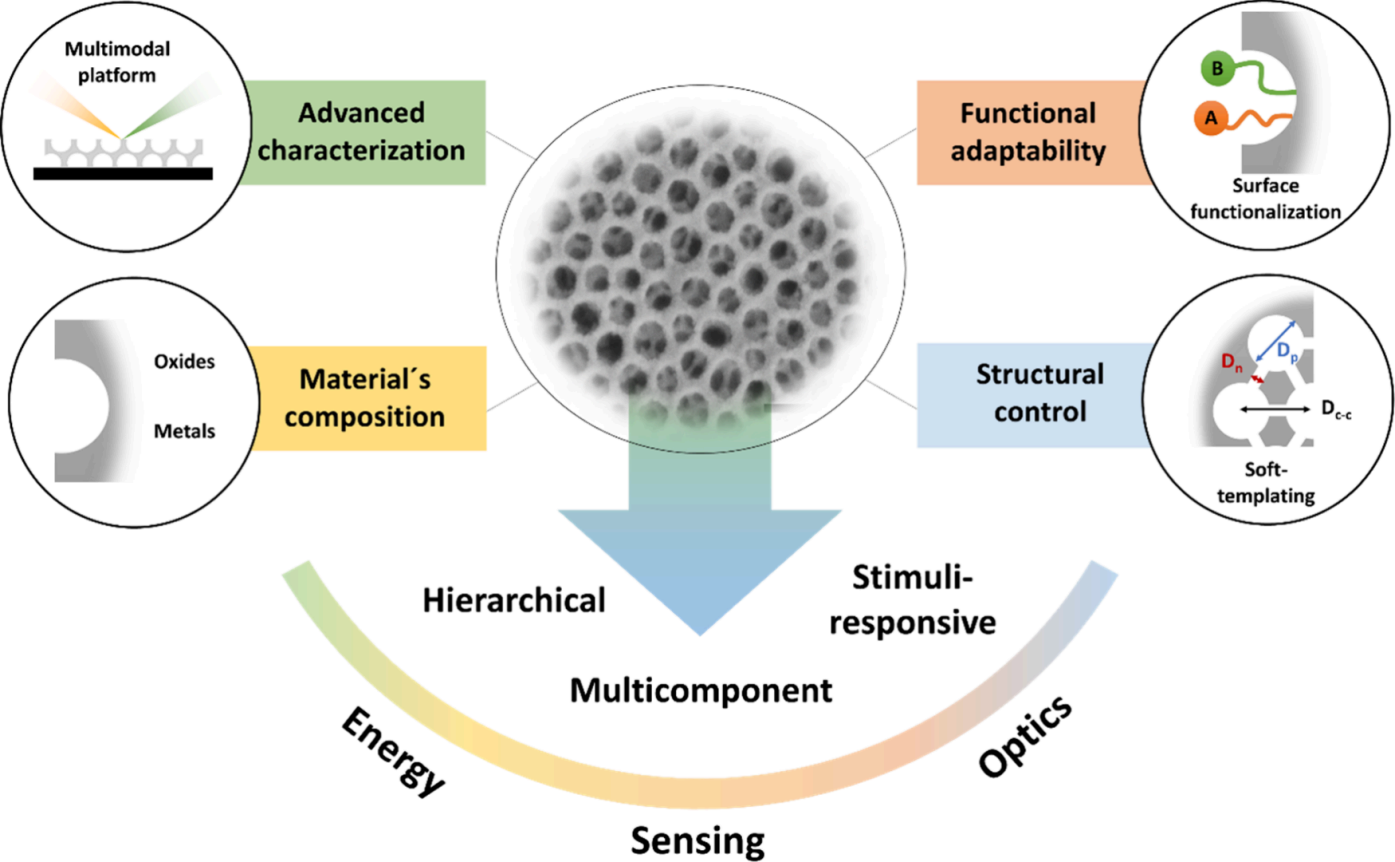
Schematic representation of the synergetic integration between the new methodologies for the fabrication of highly controllable mesoporous thin films, the supramolecular approach for the introduction of selective functionalities, and advanced characterization platforms.

## 2 From Simple to Complex: Hierarchical and Multicomponent Structures

Our work over the past decade has aimed at addressing one of the central challenges in mesoporous thin film science: how to move from simple, periodic structures toward more complex, hierarchical, and multifunctional materials, without sacrificing precision or processability. The conventional route for producing mesoporous thin films has relied on the well-established evaporation-induced self-assembly (EISA), wherein supramolecular templates and inorganic building blocks self-organize during solvent evaporation to form hybrid mesophases.^21^ There is ample evidence that the most favorable conditions imply the concerted assembly of small, hydrophilic precursors with low condensation degree, around a liquid crystalline arrangement, forming a structured hybrid mesophase. Subsequent stabilization and thermal treatment yield porous structures with precise control over pore size and connectivity. Recently, coarse-grained simulation models have provided quantitative insight into how synthetic and processing variables drive mesoscale organization.^22^ This has inspired new synthetic pathways that use preformed nanobuilding blocks with finely tuned sizes, enabling the creation of unconventional mesoporous architectures and expanding the design space.^23^

A key development in this context is the engineering of hierarchical porosity, which is defined as the integration of distinct pore regimes, typically mesopores (2–50 nm) with larger macropores (>50 nm), within a single film. While conventional strategies use binary templating methods (e.g., block copolymers (BCPs) for mesopores and colloids or phase-separation agents for macropores),^24^ such equilibrium-based self-assembly processes inherently suffer from structural parameter coupling. Thus, for example, changes to pore size also affect wall thickness, ordering, or connectivity. In our work, we have explored nonequilibrium, kinetically controlled processes, such as solvent vapor annealing (SVA) and persistent micelle templating (PMT), to decouple these parameters.

For instance, we have implemented SVA in BCP-inorganic hybrid films to selectively swell the micelles cores prior to sol-gel inorganic precursors condensation reaction.^25^ By careful tuning solvent selectivity and exposure conditions, we can reproducibly expand pore diameters while preserving morphology and order (Figure 2A). This allows postdeposition tuning without requiring new template synthesis, making the process flexible and scalable.^25^ Complementary, we have extensively developed the PMTs method as a reliable approach to effectively decouple pore size and wall thickness.^26^ In contrast to thermodynamically driven assembly, PMTs rely on maintaining kinetically “frozen” micelle structures by operating under high χN regimes or using vitrifiable core blocks.^27–29^ When combined with controlled homopolymer swelling, PMT allows for independent tuning of wall thickness and pore diameter, something rarely achievable in equilibrium systems.^30^ To date, our PMT approach is easy to execute in capital-constrained environments,^27^ simple to validate with a SAXS model, and titration series have demonstrated as many as 50 members to a sample series having as small as ∼2 Å changes in average wall thickness between samples.^28^ This level of independent tuning is also critical for fabricating mesoporous films capable of accommodating additional functional layers or guest species.

**Figure 2:**
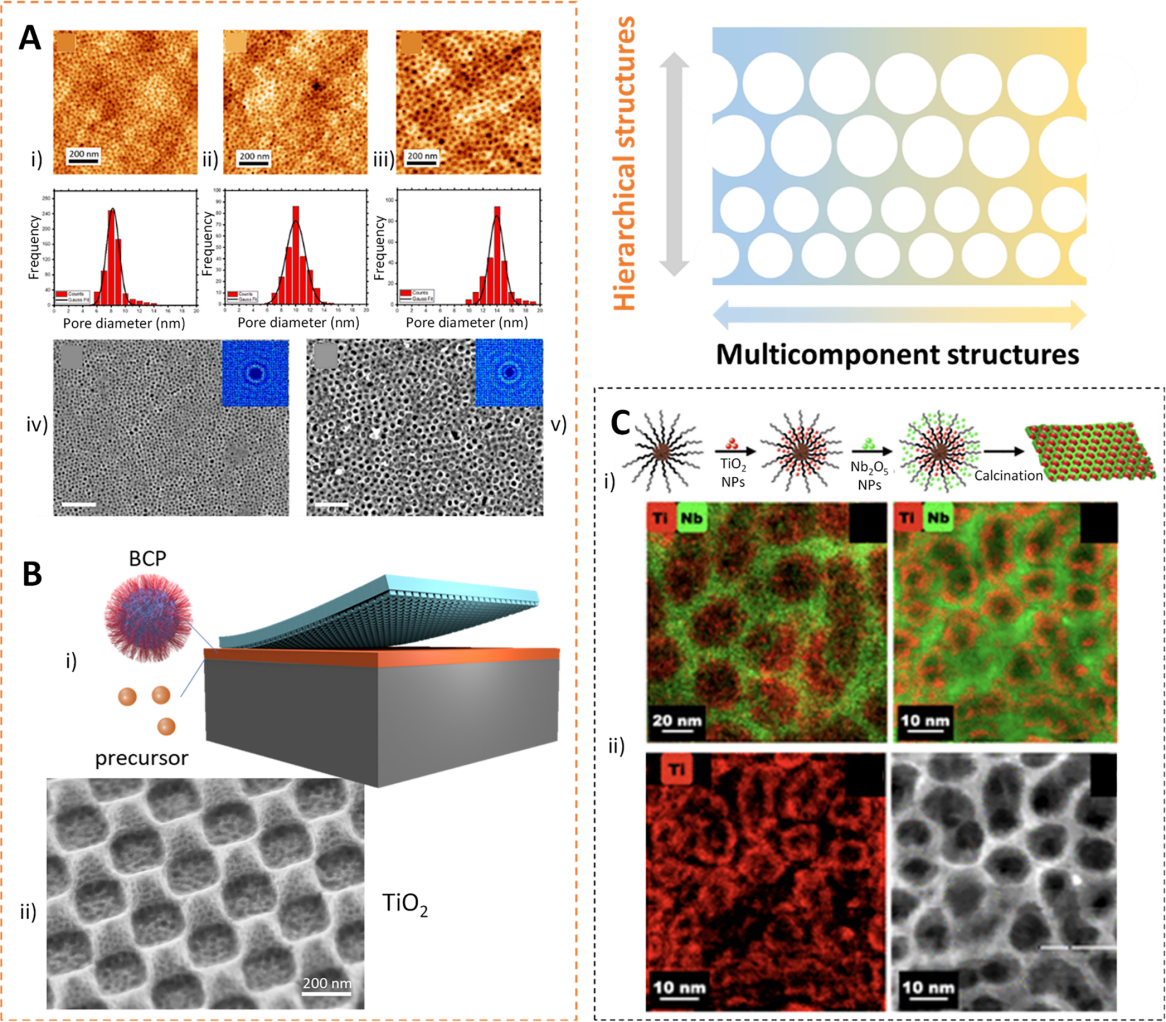
A) AFM topographical images of the TiO_2_ mesoporous films obtained at different times of SVA (0 h (i), 30 min (ii), and 1 h (iii)) and corresponding pore diameter histograms. SEM images of the TiO_2_ before (iv) and after (v) SVA annealing treatment. Adapted with permission from ref 25. Copyright 2022 American Chemical Society. B) Schematic of the direct fabrication of hierarchical inorganic porous patterns by nanoimprinting lithography (i). SEM images of the hierarchical structures created following this approach (ii). Adapted with permission from ref 31. Copyright 2013 American Chemical Society. C) Schematic process for the obtention of mesoporous heterostructures following a one-pot self-assembly process (i). STEM and STEM-EDS images of the heterostructures created (ii). Adapted with permission from ref 38. Copyright 2024 American Chemical Society.

Another important feature to consider when designing hierarchically structured films is the degree of ordering at multiple length scales. Indeed, periodic and well-organized hierarchical architectures can impart additional functionalities, such as light diffraction or anisotropic wetting behavior that cannot be obtained with “random” structures. We have found nanoimprint lithography (NIL) particularly effective for combining bottom-up and top-down strategies.^31^ As shown in Figure 2B, the process begins with a hybrid film composed of inorganic precursors and BCP micelles, which is still uncondensed and mechanically deformable. We then use a patterned elastomeric mold to imprint a defined structure onto the hybrid layer, followed by thermal treatment that induces condensation and template removal. The resulting film features a dual-scale patterning, mesoporosity from self-assembly, and larger topographical features from the stamp. This method has been successfully applied to a variety of inorganic materials, including metal oxides and mesoporous metallic films.^32,33^

Another way to obtain periodic and ordered hierarchically structures into mesoporous films is the controlled crack engineering that do not require micro- nanofabrication but is a self-assembly process (i.e., driven by thermodynamics).^27^ This approach relies on aqueous inks containing inorganic precursors and engineered polymeric latexes, which self-organize into periodic crack patterns as the solvent evaporates during deposition techniques such as dip-coating or blade-coating. Rather than being defects, these crack patterns act as intrinsic patterning elements, introducing structural control at multiple length scales without the need for external lithography. By optimizing solvent composition, drying conditions, polymer properties, and even applying external light, it is possible to precisely tune the crack size, orientation, and periodicity, offering a scalable and versatile approach to refining mesoporous architectures. More broadly, the periodic arrangement of the patterns (made either by nanoimprint lithography or crack self-assembly) has enabled the development of advanced functional films, such as antifogging metasurfaces,^34^ etching masks for fabricating solar cells,^32^ and coatings with giant anisotropic wetting properties for dew water harvesting.^35^

In addition to spatial structuring, we have prioritized efforts to integrate multiple functional components into mesoporous frameworks. Traditionally, this has involved postsynthetic loading of nanoparticles or biomolecules into preformed mesopores.^36,37^ However, such methods often suffer from poor dispersion, clogging, or weak interaction between components. In response, our groups have developed one-pot assembly methods using ABC triblock terpolymer structure-directing agents, which provide chemically distinct blocks for targeting specific nanoparticle (NP) populations during the coassembly process.^38^

We have recently developed two distinct modalities that demonstrated this concept. In one, the polymer incorporated monomer chemistries for both persistent and dynamic interactions with NPs to guide the selective deposition of different inorganic species.^30^ Persistent binding motifs, such as phosphonic acid groups, form strong, covalent-like interactions that anchor the first inorganic material added (NP_1_) permanently to the polymer. In contrast, the second material added (NP_2_) associates with the remaining hydrophilic segments via dynamic interactions from poly­(ethylene oxide) (PEO)-like blocks. In this way, only one material (NP_1_) is uniquely expressed at the surface of the pores when self-assembling layered heterostructures (Figure 2C).^38^ In a complementary approach, poly­(hydrophilic-*b*-lipophilic-*b*-fluorophobic) structure-directing agents (SDAs) exploit the orthogonality of hydrophilic and fluorophobic interactions to achieve a similar level of spatial precision over two NP populations.^39^ Here, gold nanoparticles with fluorophobic ligands selectively localize within the fluorophobic micelle cores, while the hydrophilic PEO segment directs hydrophilic oxide NPs to the micelle periphery. These methods enable the independent spatial control of two distinct NP species in two specific regions, permitting the direct self-assembly of heterostructured mesoporous thin films.

This combination of strategies marks a turning point in the field, enabling not only the fine-tuning of pore characteristics but also the integration of diverse functionalities within a single material platform. This level of control has long been a major goal in mesoporous materials research, and its realization promises to unlock a range of innovative applications, as we will describe in the following sections.

## 3 Responsive Mesoporous Thin Films: A New Frontier

A major shift in the field of mesoporous thin films has occurred in recent years, moving from their classical use as passive high-surface-area scaffolds to active components capable of real-time functional response. Unlike micro- or nanoporous systems, mesoporous thin films offer a unique balance of accessible pore volume and precisely controllable confinement on the 5–50 nm scale, ideal for incorporating functional molecules while maintaining mass transport of fluids in close contact with a tunable surface.

Over the past decade, our research groups have explored multiple routes to introduce responsiveness into these materials. The most established approach relies on molecular functionalization. Inspired by biological membranes, we have grafted stimuli-responsive components, such as temperature-, pH-, or ion-sensitive moieties, directly onto the pore walls to regulate mass and charge transport, effectively mimicking gating behavior seen in natural ion channels. While similar gating mechanisms also occur in polymer membranes or hydrogels, the mesoporous framework offers superior structural rigidity and long-term operational stability, enabling reproducible on/off control even under nanoconfined and cycling conditions.

For instance, mesoporous thin films functionalized with low LCST polymers such as poly­(*N*-isopropylacrylamide) (PNIPAM) exhibit well-defined thermal responsiveness.^40^ Below the lower critical solution temperature (LCST), PNIPAM chains are swollen and hydrated, extending into the mesopores and effectively limiting the passage of ions and molecules. Upon heating above the LCST, the chains collapse onto the pore walls, reopening the channels and boosting ion conduction (Figure 3A). Electrochemical measurements confirm that these changes are fully reversible and withstand cycling, producing distinct low and high-conductance states that closely mimic the temperature-dependent gating mechanisms seen in biological ion channels.^41^

**Figure 3:**
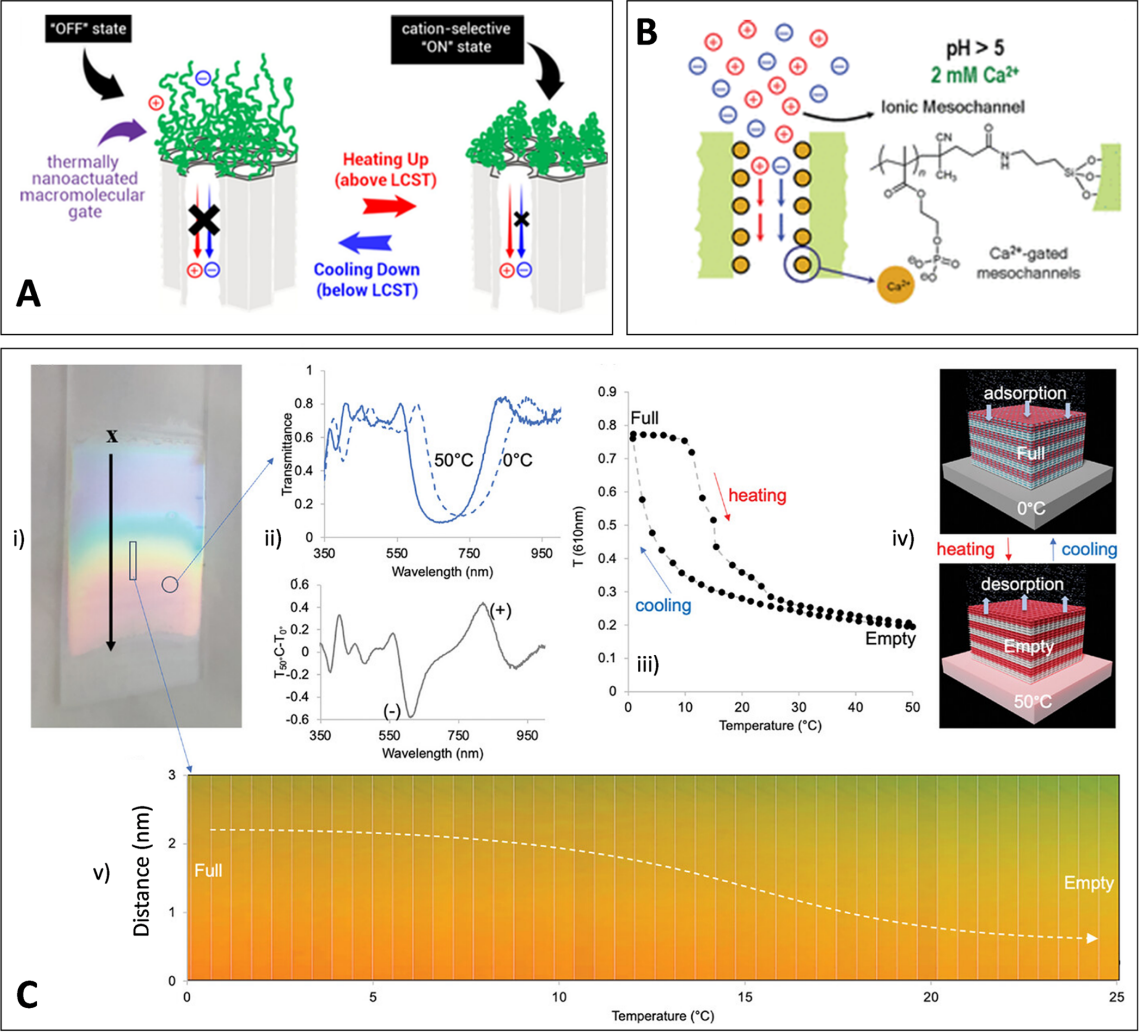
Representative examples of stimuli-responsive mesoporous thin films, illustrating different activation mechanisms based on both molecular and functional responsiveness. A) Thermally triggered ion gating in PNIPAM-grafted mesoporous thin films, switching between “off” and “on” states across the LCST. Adapted with permission from ref 40. Copyright 2017 Wiley-VCH Verlag GmbH. B) Chemically gated mesochannels activated by Ca^2+^ binding and pH-dependent conformational changes of phosphate-bearing brushes. Adapted with permission from ref 41. Copyright 2012 American Chemical Society. C) Functional responsiveness of mesoporous photonic films integrating thermal-regulated adsorption/desorption dynamics: (i) photograph showing color variation under a thermal gradient; (ii) transmittance spectra at 0 and 50 °C; (iii) Bragg peak shift during heating and cooling cycles; (iv) schematic of thermally driven adsorption/desorption; (v) spatiotemporal map showing vertical pore filling evolution as a function of temperature. Adapted with permission from ref 45. Copyright 2025 The Authors.

In addition to thermal actuation, we have also developed chemically gated systems. For example, phosphate-bearing polymer brushes embedded within the pore walls undergo protonation or calcium ion binding depending on the local environment, leading to reversible modulation of ion transport.^41^ At low pH, phosphate groups become protonated, lowering the surface charge and closing the mesoporous channels. At higher pH or in the presence of divalent cations, the brushes deprotonate or reorganize, reopening the pores (Figure 3B). These changes enable selective transport, acting as biomimetic analogs to ligand-gated ion channels. Using both experiment and modeling, we have shown how polyelectrolyte length and charge density influence permselective transport, and how the interplay between confinement and ionic atmosphere alters brush configuration and accessibility.^42^ These systems provide precise, tunable control over ionic flux under operational conditions relevant to biosensing and drug delivery.

Beyond molecular strategies, our work has also demonstrated that mesoporous thin films can exhibit functional responsiveness, where structural or environmental changes are directly transduced into physical signals. For instance, by depositing mesoporous oxides onto Bragg reflectors, we have created systems that support coherent phononic resonances up to 100 GHz.^43^ These high-frequency acoustic modes are directly modulated by pore infiltration with liquids, gases, or biomolecules, offering label-free, nanoscale acoustic sensing. Extending this concept to gradient-porosity stacks promises tunable phononic filters and high-Q resonators whose response can be modulated by chemical or thermal inputs.^44^ Similarly, we have demonstrated that mesoporous architectures can also support autonomous homeostatic devices. For instance, mesoporous photonic crystals integrating a vapor-regulated feedback mechanism allow films to autonomously adjust color and transmission under changing humidity or solar flux (Figure 3C).^45^

In another example, TiO_2_-based mesoporous thin films were used to mediate directional chemical communication between droplets placed on their surface.^46^ In these systems, two adjacent droplets charged with a reagent (H_2_O_2_) and a catalyst (KI) on the nanoporous surface interact in a “vectorial” (i.e., one-way) manner: when the respective liquids come into contact, the peroxide-containing droplet extends a finger-like projection that connects to the KI-containing droplet. This behavior, driven by channeling the chemical reaction (i.e., peroxide decomposition) and the resulting chemical gradients, mimics simple cellular processes like engulfing. These findings illustrate how mesoporous systems can transcend static responsiveness, enabling programmable spatiotemporal interactions across interfaces. As a closing note to this section, we see these developments not simply as enhancements of classical transport control but as early demonstrations of how mesoporous materials can support self-organized behaviors that emerge from spatial organization and confinement, opening promising paths toward autonomous chemical devices, microreactors, or logic elements in soft robotics and microfluidics.

## 4 Mesoporous Thin Films for (Bio)Sensing: New Directions

Alongside our exploration of responsive functionalities, we have increasingly focused on translating mesoporous thin film architectures into high-performance platforms for (bio)­sensing. While porous silicon and anodic aluminum oxide (AAO) membranes laid the groundwork for porous-based biosensors due to their high surface areas and well-ordered pore structures, they come with inherent limitations. AAO, for instance, are typically fabricated as free-standing membranes that must be manually placed on top of the electrode surface. Similarly, porous silicon often suffers from less structural control and requires harsh etching steps during fabrication, which can compromise reproducibility and biocompatibility.

By contrast, the solution-based self-assembly methods we have developed allow the direct modification of electrode surfaces via casting and deposition processes, enabling seamless integration into device fabrication workflows. These methods also offer quantitative control over pore size, channel connectivity, and thickness. Moreover, integrating stimuli-responsive materials, such as the ones shown in Section 3, to the mesoporous framework could further refine control over analyte transport and signal transduction, ultimately leading to sensor platforms with significantly improved selectivity and sensitivity.^2^

Focusing on specific applications, our work has shown that mesoporous thin film architectures are particularly well-suited for enzyme immobilization. Compared to flat or polymeric supports, mesoporous films offer size-selective confinement, high surface-to-volume ratios for enzyme loading, and enhanced operational stability. These features make them ideal for preserving enzymatic function in confined environments, while also supporting sensor reusability and longevity.

One specific application of these is in the development of electrochemical glucose sensors, where enzyme leaching and activity loss remain key challenges. In our system, we addressed these issues by employing ordered mesoporous aluminosilicates functionalized with amino groups, which introduce positive surface charges that interact favorably with the negatively charged glucose oxidase (GOx) at physiological pH.^47^ This strategy results in higher enzyme loading, prolonged catalytic activity, and enhanced sensitivity and reproducibility, demonstrating how rational interface engineering within a mesoporous matrix can directly translate into improved biosensing performance (Figure 4A). We have also explored mesoporous TiO_2_ thin as carriers for enzymes such as organophosphate hydrolase (OPH), where the material’s inherent chemical robustness (resisting pH variations from ∼ 2 to 12) protects the enzyme while maintaining substrate diffusion through well-defined channels.^36^ This dual approach, enhancing enzyme retention and activity through tailored surface chemistry, offers a clear advantage over disordered porous supports or nanoparticle-based films, which lack the same degree of structural precision or integration potential, resulting in robust sensor platforms that are both reusable and reliable, whether for continuous glucose monitoring in diabetic care or for environmental detection of toxic agents.

**Figure 4:**
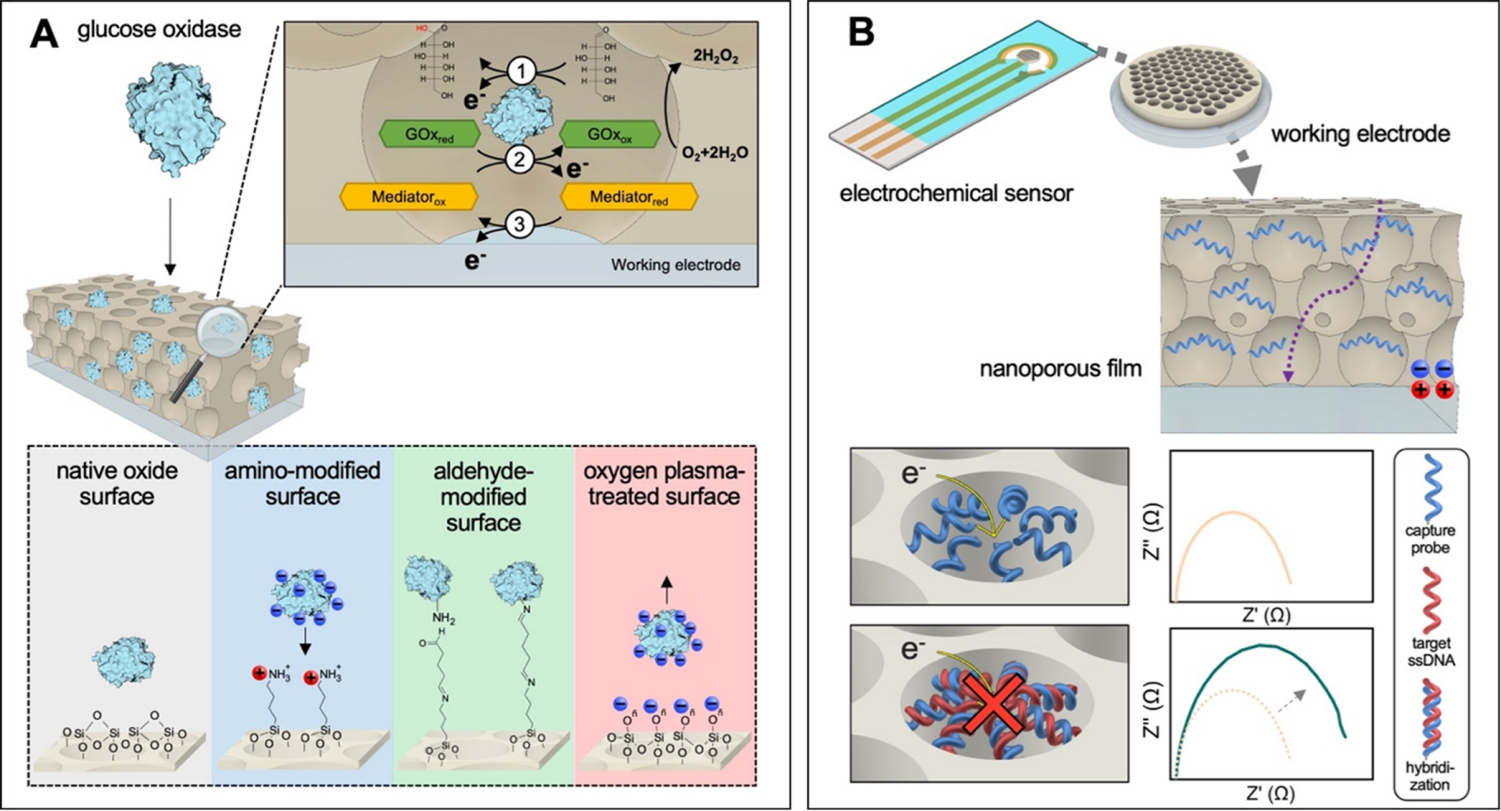
A) Schematic representation of enzyme immobilization in mesoporous films via native oxide, amino-modified, aldehyde-modified, and oxygen plasma-treated surfaces. The enzyme catalyzes glucose oxidation, generating an electrochemical signal for glucose detection. Adapted with permission from ref 47. Copyright 2023 The Authors. B) Illustration of an ultrasensitive DNA detection platform based on nanopore blockage-based electrochemical sensing. Adapted with permission from ref 48. Copyright 2025 Royal Society of Chemistry.

Another direction of our research has focused on the development of electrochemical deoxyribonucleic acid (DNA) biosensors using mesoporous thin film electrodes. While traditional detection methods such as polymerase chain reaction (PCR) remain highly sensitive, their complexity and instrumentation requirements limit use in decentralized settings. In contrast, using BCP-templated mesoporous films, we have fabricated label-free DNA sensors that achieve femtomolar detection limits through an impedance-based readout mechanism.^48^ By tuning pore size to match oligonucleotide dimensions and ensuring uniform probe distribution, we create platforms where DNA hybridization produces reproducible and quantifiable changes in ionic transport through the film (Figure 4B). Notably, these devices deliver accurate results in less than 20 min, making them ideal for point-of-care diagnostics and pathogen detection in low-resource environments, two applications where low power, rapid signal transduction, and high specificity are paramount.

Beyond electrochemical sensing, we are also developing mesostructured SERS substrates for highly sensitive molecular fingerprinting. Using the multicomponent templating strategies described in Section 2, we have fabricated mesoporous oxide thin films loaded with plasmonic nanoparticles (e.g., Ag or Au) whose positioning and confinement are precisely controlled.^36,50^ Unlike drop-cast or aggregated nanoparticle films, which can suffer from hot spot variability and poor reproducibility, our approach ensures uniform electromagnetic enhancement, enabling reproducible SERS signals across large sample areas.^52^ In this context, the one-pot fabrication methodologies discussed in Section 2, which enable the controlled positioning of plasmonic nanoparticles, functional moieties, and matrix components within a single fabrication step, hold great promise for further enhancing the performance and structural complexity of this type of sensor.

Looking ahead, we believe that the next frontier lies in designing adaptive, multifunctional sensing platforms that combine stimuli-responsiveness, selective molecular recognition, and real-time signal modulation, all embedded within a single mesostructured framework. The ability to colocalize biological recognition elements, responsive chemistries, and electronic or optical transducers in a scalable thin film format opens up exciting possibilities for implantable diagnostics, wearable health monitors, and smart environmental sensing surfaces. In our view, mesoporous thin films are uniquely positioned to meet these demands, provided that ongoing efforts continue to expand their functional complexity while maintaining integration compatibility. We see this as a defining challenge for the field and a core direction for our future work.

## 5 Tailored Mesoporous Thin Films for Advancing Energy Applications

In energy-related applications, materials are often required to strike a balance between high-rate charge transport and long-term operational stability. This is particularly true in systems such as electrochemical storage devices or catalytic reactors, where fast ion mobility, accessible surfaces, and structural robustness must all coexist. Mesoporous thin films offer a way to engineer this balance by providing controlled pathways for diffusion while maintaining the spatial confinement necessary to probe transport or reaction dynamics directly.

Our research has focused on exploiting this confinement to study and improve intercalation-based energy storage, as well as to develop scalable routes toward mesostructured catalytic materials. By combining methods such as persistent micelle templates and polymer-assisted reduction, we have explored how systematic variations in wall thickness, crystallinity, and chemical composition influence both performance and underlying mechanisms. In the following sections, we present selected examples where mesoporous thin films have served as both functional components and well-defined model systems to understand rate-limiting processes, durability, and catalytic activity in energy-relevant environments.

A central focus has been the study of intercalation pseudocapacitance, a charge storage mechanism where faradaic reactions exhibit surface-limited kinetics.^53^ First reported in 2013,^51^ this process combines battery-like energy density with capacitor-like rate capability, enabling high energy density and (dis)­charge times on the order of seconds. The type of rate-limiting processes can be evaluated by cyclic voltammetry using

1 $$i=a\upsilon^{b}$$

where *i* is the peak current, *v* is the voltage sweep rate, and *b* is an exponent indicating the dominant mechanism. A *b*-value near 1 suggests surface-limited (capacitor-like) behavior, while *b*-values near 0.5 reflect diffusion-limited (battery-type) kinetics.

To investigate how the mesostructure affects such behavior, we developed isomorphic sequences of PMT-based nanomaterials mesoporous films with constant pore size and varying wall thickness.^52^ This design enables the systematic investigation of how electron transport length, electrolyte diffusion resistance, and intercalation path length affect the electrochemical response (Figure 5A). In mesoporous T-Nb_2_O_5_ thin films, we introduced the “surface-limited threshold” (ν_slt_), defined as the maximum scan rate with surface-limited behavior (b ≈ 0.9). Films with thinner walls retained surface-limited behavior to higher ν_slt_ values, while films with thicker walls transitioned to diffusion-limited kinetics at lower ν values. These trends were consistent with Fickian scaling and confirmed that intercalation length is a key determinant of rate behavior.^52^ Follow-up studies introduced an “insert-intercalate” model,^53^ that removed a popular assumption of a special near-surface layer, and enabled much improved fits of the voltage sweep-rate dependent *b*-values (Figure 5B). This clarified how mesoporous architectures can be rationally tuned to shift the balance between surface and bulk-controlled processes.

**Figure 5:**
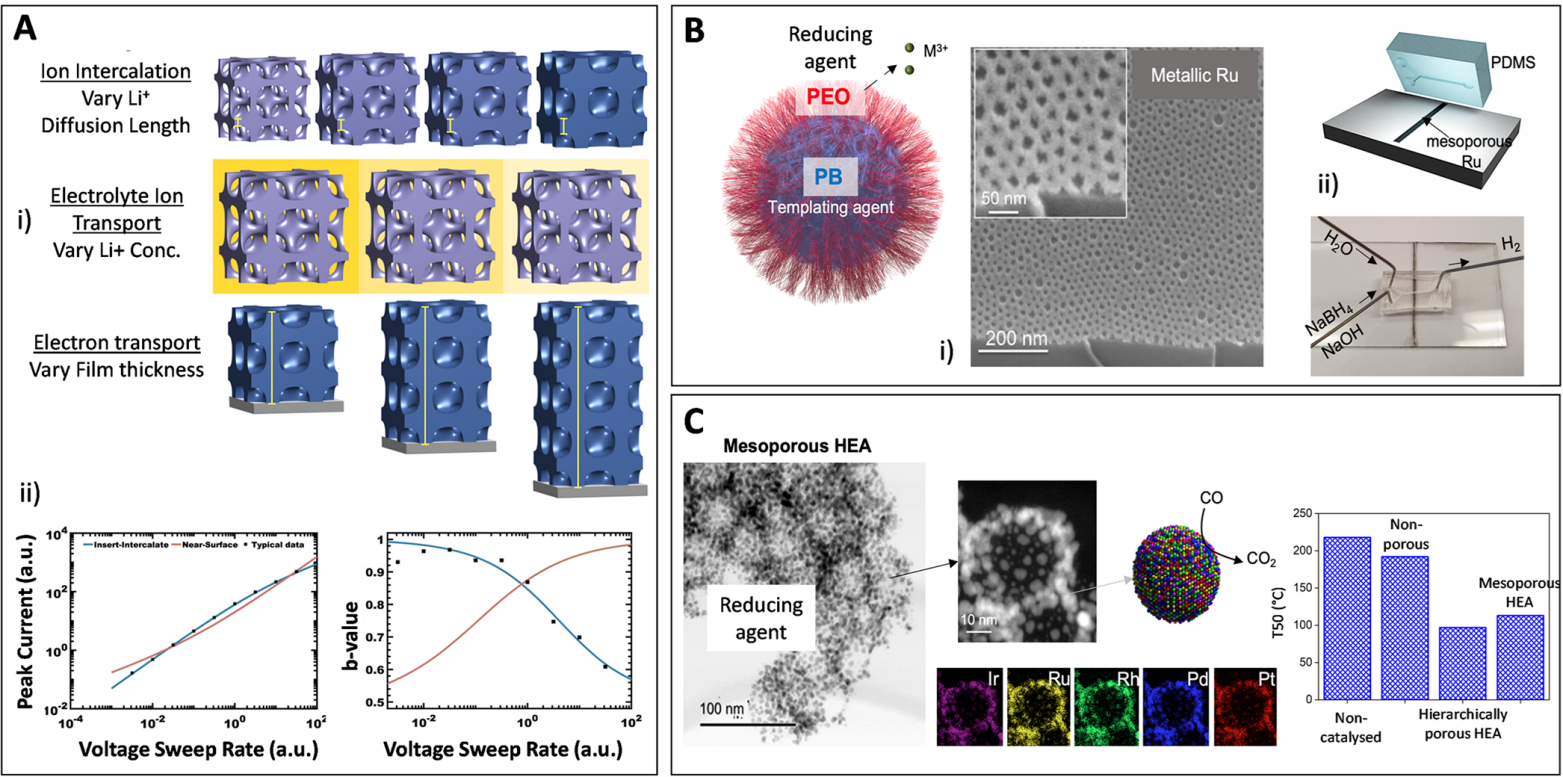
A) Mesoporous materials prepared using persistent micelle templates enable electrochemical interrogation of cause-and-effect by probing the effects of changing each transport process, including ion intercalation, electrolyte diffusion, and electron transport (i). Single-variable experiments are ideal to unravel complex and convolved processes that are often found in electrochemistry. For example, the rapid intercalation of lithium into diverse materials is often studied with cyclic voltammetry, where the voltage sweep rate dependence of the *b*-value recently helped distinguish between competing mechanistic models (ii). B) Micelles can be used not only as templates but also as reducing agents to obtain metallic mesoporous films. Ru mesoporous films, as shown in the SEM image (i), can be integrated into microfluidic reactors to promote hydrogen generation reactions (ii). Adapted with permission from ref 56. Copyright 2021 Wiley-VCH GmbH. C) The carbo-reduction strategy can be expanded toward macro- and mesoporous HEAs, as shown in the TEM micrographs. Adding porosity to HEA materials promotes the catalytic CO oxidation. Adapted with permission from ref 57. Copyright 2022 American Chemical Society.

Beyond geometry, we also investigated how wall crystallinity affects transport.^54^ Mesoporous Nb_2_O_5_ films with average pore diameters of ≈95 nm were calcined at temperatures between 300 and 600 °C to produce a continuum of wall structures ranging from highly amorphous to crystalline. Applying the insert-intercalate model, we found that partial amorphization of otherwise crystalline walls boosts the solid-state diffusion by 12.2% while simultaneously amplifying surface-reaction limitations by 17.0%. Consequently, these optimally disordered films maintain 95% of their lithiation capacity at ∼800 mV/s (1,600 C equivalent), demonstrating that precise control over wall disorder and crystallinity provides a direct means to tune power delivery in mesoporous oxide electrodes. More recently, the same material system achieved 90.8% capacity retention after 250,000 cycles,^55^ demonstrating their long-term electrochemical stability.

We have also extended these approaches toward catalytic applications by tailoring the chemical composition of mesoporous networks. In particular, we have developed a synthetic approach in which the BCP micelles can be used not only as structure-directing agents but also as reducing environments.^56^ Using poly­(butadiene)-block-poly­(ethylene oxide) (PB-*b*-PEO) micelles, we demonstrated that the PEO corona reduces metal precursors via in situ formation of metastable metal-carbonyl complexes, while the PB core templates porosity. Thermal treatment under inert conditions yields mesoporous noble-metal films, such as Ir, Ru, or Rh, without requiring external reducing agents. This process is solution-based, scalable, and adaptable to various substrates.

This polymer-assisted approach is also compatible with soft NIL, enabling the fabrication of hierarchically structured mesoporous metallic films (Figure 5B). For instance, mesoporous ruthenium-based films, prepared by carboreduction of BCP micelles, have shown high catalytic efficiency for sodium borohydride hydrolysis, a hydrogen storage compound capable of delivering hydrogen on demand.^56^ Their high surface area and porosity support rapid hydrogen generation under mild conditions, with activity comparable to top-performing Ru catalysts, even at lower NaBH_4_ concentrations. Unlike powder-based materials, these films can be integrated into microfluidic reactors for continuous hydrogen production at miniaturized scales, particularly advantageous for multistep processing or reactive species handling.^56^

Most recently, we extended the method to fabricate hierarchically structured mesoporous high entropy alloys (HEAs),^57^ a class of materials with increasing relevance in electrocatalysis. By using poly­(styrene) (PS) or poly­(methyl methacrylate) (PMMA) particles and reducing templates, we synthesized hierarchically structured macro and mesoporous HEAs,^59^ whose walls were composed of small nanocrystals in which five different Pt-group metals are homogeneously distributed.^57^ These materials demonstrated high activity for CO oxidation, a benchmark catalytic reaction, and benefited from reduced activation temperatures due to their hierarchical porosity and accessible surface area (Figure 5C).

Despite these advances, key challenges remain. For intercalation-based systems, mesoporous thin films still face trade-offs between wall thickness, crystallinity, and pore connectivity, especially as one moves toward multivalent ions or lower operating potentials. Thinner walls improve rate capability but can compromise structural integrity over repeated cycling; higher crystallinity improves conductivity but can reduce diffusivity. These relationships are well understood in theory but remain difficult to optimize simultaneously in practice. For catalytic applications, a persistent limitation lies in the chemical complexity of multicomponent films. Although our polymer-assisted reduction methods allow access to noble-metal alloys and even high entropy compositions, controlling their surface composition, oxidation state, and spatial distribution within hierarchical structures is far from trivial. Most current approaches still rely on indirect indicators of uniformity and activity, and a more complete mechanistic picture is needed to guide rational design.

We believe that addressing these challenges will require closer coupling between synthesis and operando characterization, as well as refined model systems that isolate individual structural variables without oversimplifying the material system.

## 6 Mesoporous Thin Films: What Is Next?

This Account has traced the work developed in our research groups over the past decade, focused on pushing mesoporous thin films beyond their classical roles as high-surface-area scaffolds, toward active components in responsive, multimodal devices. This shift is not only driven by improvements in fabrication or functionalization, but by a growing understanding that spatial confinement, mechanical coupling, and dynamic gating can be exploited to program entirely new material behaviors. As this transformation unfolds, the central challenge becomes one of integration: how to translate confined structure, chemical complexity, and dynamic behavior into systems that perform reliably and autonomously in real-world settings.

Several recent developments point to what this integration might look like: mesoporous films operating as nanoscale sensors, homeostatic photonic devices, or ionic gates. But to support this new functionality, characterization methods must evolve accordingly. Many of the processes involved, including ion movement, wetting dynamics, structural relaxation, and dynamic gating, are all occurring at nanometer and nanosecond scales, where conventional characterization tools fall short.^58^ Quasielastic neutron scattering (QENS) and related neutron spectroscopies excel in this regime, delivering diffusion coefficients, jump lengths, and relaxation times for confined species. For instance, QENS studies of single-chain polymer nanoparticles have mapped how varying degrees of confinement alter segmental relaxation times and activation energies,^59^ directly demonstrating that nanoscale restriction can either slow down or accelerate molecular motion.^60^ Future applications of neutron spectroscopy in mesoporous thin films could include monitoring lithium diffusion across pore walls during cycling, or mapping gating kinetics in situ within hybrid organic-inorganic networks. Coupling such data with structural inputs from small-angle scattering and vibrational spectroscopies may provide the mechanistic understanding needed to bridge design and function.

In parallel, EP is evolving into a more versatile and informative characterization tool. As noted in the Introduction, new probe molecules and refined modeling approaches have extended its capacity to capture pore size distributions, internal contact angles, and surface energies even in hierarchical or functionalized architectures. However, the next step will be to move toward operando EP, capable of tracking transient changes in porosity, wetting, or surface chemistry during stimuli exposure or device operation. This demands the development of flow-compatible, time-resolved measurement platforms, as well as models that account for dynamic, nonequilibrium conditions. Such tools will be crucial for evaluating the real-world performance of mesoporous systems, particularly those used in sensing, gating, or energy conversion.

At the same time, we see continued value in rethinking synthetic approaches, not only in terms of structure, but also in chemical versatility. One particularly promising direction lies in the modular assembly of mesoporous walls from functional homopolymers equipped with end groups such as azides or alkynes for click chemistry. This would enable the creation of compositional libraries in which polymers with specific ionic, hydrophobic, catalytic, or bioactive properties are assembled in configurable sequences. Such “à la carte” synthesis could decouple the generation of porosity from the design of wall functionality, offering a powerful route to tailor-made materials with predictable, programmable responses. Moreover, we think that the convergence of top-down and bottom-up fabrication approaches, long treated as parallel but distinct paths, will continue to be essential. Examples discussed here, from crack-guided self-assembly to nanoimprint-patterned mesostructures, demonstrate how integrating lithography with molecular self-assembly enables hierarchical control over structure, orientation, and functionality in a single platform. This integrative paradigm will be particularly critical in fields such as electrochemical energy storage, where fast ion transport, mechanical resilience, and interfacial stability must all be addressed simultaneously.

With these advances, mesoporous thin films are well-positioned to evolve into a new class of adaptive materials, ones that do not just respond to their environment, but help define it. The tools are in place. The next steps will define how far this architecture can go.
